# Plectin-Mediated Intermediate Filament Functions: Why Isoforms Matter

**DOI:** 10.3390/cells10082154

**Published:** 2021-08-21

**Authors:** Gerhard Wiche

**Affiliations:** Max Perutz Laboratories, Department of Biochemistry and Cell Biology, University of Vienna, 1030 Vienna, Austria; gerhard.wiche@univie.ac.at

**Keywords:** plectin, isoforms, intermediate filaments, mechanotransduction, actomyosin, microtubules

## Abstract

This essay focuses on the role of plectin and its various isoforms in mediating intermediate filament (IF) network functions. It is based on previous studies that provided comprehensive evidence for a concept where plectin acts as an IF recruiter, and plectin-mediated IF networking and anchoring are key elements in IF function execution. Here, plectin’s global role as modulator of IF functionality is viewed from different perspectives, including the mechanical stabilization of IF networks and their docking platforms, contribution to cellular viscoelasticity and mechanotransduction, compartmentalization and control of the actomyosin machinery, connections to the microtubule system, and mechanisms and specificity of isoform targeting. Arguments for IF networks and plectin acting as mutually dependent partners are also given. Lastly, a working model is presented that describes a unifying mechanism underlying how plectin–IF networks mechanically control and propagate actomyosin-generated forces, affect microtubule dynamics, and contribute to mechanotransduction.

## 1. Introduction

Plectin, a cytolinker protein and member of the plakin protein family [1], was described and first characterized some 40 years ago [2]. The protein was shown to play a central role in the organization and performance of the vertebrate cell cytoskeleton. Not only is it essential for the functionality of intermediate filament (IF) networks of different types, but it also affects the dynamic behavior and site of action of the contractile and polarizing actomyosin and microtubule (MT) network systems [3,4,5,6]. With a polypeptide chain length of ~4500 amino-acid residues, plectin is a huge multidomain protein, comprising a variable *N* terminus, followed by a classical actin-binding domain (ABD) and a plakin domain, a central helical rod domain, and a long *C*-terminal domain [5]. The ABD comprises a pair of calponin homology (CH) domains, and the plakin domain contains nine spectrin repeats with an SH3 domain embedded within the fifth repeat [7]. Via their central α-helical domain, plectin molecules dimerize to form a ~200 nm long rod of a coiled-coil helical structure; the lateral association of rod domains leads to plectin oligomers. The *C*-terminal domain is built up of six plectin repeat domains (PRDs) of roughly 300 residues each, separated by linker regions of variable sizes, and a short *C*-terminal tail. Within each PRD lies a highly conserved central core, and within the linker region between PRDs 5 and 6 resides plectin’s major IF-binding domain (IFBD) [8].

What makes plectin quite unique is its isoform diversity based on multiple variable first exons alternatively splicing into a common exon 2, generating a hydra-like pattern of alternative transcripts. There are about a dozen of such transcripts, sharing a common structure composed of over 30 exons, but differing from each other by their very *N*-terminal head domains. Additional alternative splicing of small exons into plectin’s ABD and of a single large exon (exon 31) encoding the entire α-helical coiled-coil rod domain of the molecule gives rise to a multiplicity of plectin variants [9]. The isoforms encoded by these transcript variants were named according to their alternative first coding exons in chronological order of their identification. Accordingly, the first isoform identified was given the name plectin isoform 1 (P1), followed by isoforms P1a, P1b, P1c, etc., with P1k being the last isoform identified (for a more detailed description of isoforms and corresponding transcript variants, see [5]).

**Figure 1:**
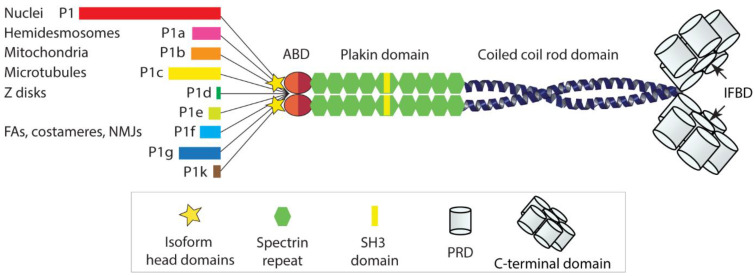
Schematic representation of a plectin dimer. The *N*-terminal domain contains two actin-binding domains (ABDs) each consisting of two calponin homology domains (light and dark red), and two plakin domains each comprising nine spectrin repeats (green) and one (noncanonical) SH3 domain (yellow). The central coiled-coil rod domain is ~200 nm long. The *C*-terminal domains each contain six plectin repeat domains (PRDs), where PRDs consist of a conserved region, referred to as module, and a linker region, one of which harbors the universal IF-binding domain (IFBD). The stars at the *N* termini of the polypeptide chains stand for the different isoform-specific head domains. Nine alternative head domains are depicted (color-coded bars), giving the name to plectin isoforms P1, P1a, P1b, etc. Major isoform targeting sites are indicated. Note: the figure is not drawn to scale, while the length of the bars is proportional to the size of the head domains.

As shown by expression profiling, different types of cells and tissues express these isoforms in different combinations and proportions, often in dependence of their developmental stage [10,11]. The observation of distinct isoform expression patterns has been the basis for a working model where the expression of different sets of plectin isoforms is tailored for particular functional needs of the cell, in a kind of “custom-made” fashion. Important for the understanding of plectin’s multiple functions was the observation that the various isoform-specific *N*-terminal head domains dictate the cellular localization of the isoform [12], by docking to distinct interaction partners. A few isoform-specific interaction partners have indeed been identified; however, in several other cases, isoform-specific interaction partners and targeting mechanisms are still unknown. As all the isoforms are endowed with a universal high affinity IF-binding domain at their *C* termini, they will recruit and anchor IF networks of any type to the various sites and structures with which they are associated. This is an important concept, as it leads to an isoform-dependent interlinking of different cellular structures and organelles via IFs, with major implications for cytoarchitecture, shape, polarization, and migration potential of cells [4,5,6,13]; see Figure 1 for a schematic drawing of the plectin molecule and its diverse head domains).

Among the cellular structures that are linked via plectin to IFs are all sorts of cytoskeletal junctional complexes, including hemidesmosomes (HDs) and focal adhesions (FAs) in epithelial cells and fibroblasts, costameres and neuromuscular junctions (NMJs) in myofibers, myelin sheath-stabilizing junctions of Schwann cells, penetrating invadopodial protrusions of metastatic cancer cells, and others (for details see [5,6,14,15]. In addition, plectin links IFs to mitochondria and the nuclear/ER membrane and builds bridges to the actomyosin machinery and microtubules (MTs). In addition to anchoring IFs, plectin dimers and oligomers can interlink IFs, thereby physically consolidating network formation. Given plectin’s multiple interaction potentials and strategic locations, it is not unexpected that its dysfunction leads to many disorders and diseases [16]. In fact, plectin has become a paradigm for a protein that, when dysfunctional, causes multisystemic diseases, where different tissues, cell types, and organs, are affected by its dysfunction; an important role of plectin is also emerging in cancer [15,16].

The type of human diseases caused by plectin loss or dysfunction, and the phenotypes of corresponding animal (mouse) models clearly indicate that plectin plays an important role in maintaining the structural and functional integrity of cells and tissues exposed to great mechanical stress, such as skin, muscle, intestine, and vasculature. Moreover, there is mounting evidence that plectin’s IF-coupling function is essential for mechanotransduction and cell motility. Given plectin’s multiple interaction potentials and functional repertoire, a central question arising is whether there is a unifying mechanism underlying its versatile actions.

Indeed, in light of recent work (see below) establishing the ability of IFs to form viscoelastic networks that absorb and dissipate energy, thereby counteracting actomyosin forces and MT penetration, the concept of plectin isoform-mediated targeting and IF recruitment has gained new momentum. IF recruitment to sites where buffering capacity is needed, such as cytoskeletal junctions, would provide an efficient mechanism for transducing mechanical signals coupled with local counterbalance of cytoskeleton-generated forces. Previously established and new evidence in support of such a mechanism is presented here along with various other aspects with relevance to plectin’s proposed role as mediator of essential IF functions.

## 2. IFs and Plectin as Modulators of Mechanical Cell Properties

The concept of isoform-dependent interlinking of different cellular structures and organelles via IFs receives support from recent studies providing new insights into the mechanisms how IFs modulate biomechanical cell properties. Individual IFs were shown to be highly resistant to disruption upon stretching and, at a certain point, to undergo strain-stiffening [17,18]. Subjected to experimental setups that enabled their cyclic stretching and relaxation, filaments were shown to absorb and dissipate over 70% of the input energy [19]. Similar elastic and strain-stiffening properties were found to apply to IF networks [20]. Thus, contrary to networks formed of actin or MTs, IF networks are highly elastic. When composite networks of vimentin and actin were studied, actin was found to decrease the viscosity of IFs even in the absence of any crosslinker, and, at high strains, the actin network softened, whereas the IF network stiffened [20]. A number of studies performed on living cells are consistent with these in vitro data. The vital character of keratin filaments for biomechanical cell properties as a buffer system to withstand mechanical stresses was demonstrated in studies on keratin-depleted keratinocytes [21]. Studying the mechanisms of how cells in epithelial sheets are able to withstand large deformations, Latorre et al. [22] showed that strain-softening was achieved by dilution of the actin cortex, while re-stiffening occurs via keratin filaments, as part of a mechanism called superelasticity. Similarly, using optical tweezers to perform micromechanical measurements in living cells and in IF-enriched cytoskeletons devoid of actin and MTs, a hyperelastic rubber-like vimentin IF network was shown to be essential for mechanical behavior of cells under deformations [23]. By interacting with other cytoskeletal systems and organelles, vimentin IF networks significantly enhanced cytoplasmic mechanical strength and toughness under dynamic deformations, through slowing poro/viscoelastic relaxations. Moreover, it was shown that, through its elasticity, the vimentin network can effectively propagate local stress and strain into a larger region of the cell, deforming MTs and actin filaments which interpenetrate and interact with the IF network. Thus, the vimentin IF network not only significantly enhances the strength and toughness of the cytoplasm, reducing the risk of cell damage during processes involving large deformations, but also effectively disperses locally induced mechanical stress to larger regions within individual cells, enabling the dissipation of energy throughout a cell [23].

Plectin exhibits several structural features that potentially can contribute to the viscoelastic properties and mechanosensing ability of IF networks. One of these is its plakin domain which is well conserved among proteins of the plakin and spectraplakin families, such as desmoplakin, plectin, MACF/ACF7, *Drosophila* Shot, and *C. elegans* VAB-10 [24]. The domain consists of nine spectrin repeats with a noncanonical SH3 domain embedded in their middle. A wide range of proteins containing spectrin repeats are known to mechanically unfold when cells are stretched [25]; thus, the plakin domain could provide elasticity to the molecule. SH3 domains generally mediate protein–protein interactions. However, the crystal structures of desmoplakin and plectin suggest that the SH3 domain of plakins is autoinhibited by the preceding spectrin repeat [7,26]. It took large-scale force-probe molecular dynamics simulations on two plakins, plectin and desmoplakin, to show that force unravels the spectrin repeats and abolishes the autoinhibition of the SH3 domain [27]; a similar conclusion was reached in the analysis of *C. elegans* plectin homolog VAB-10A [28]. Since the unraveling of the spectrin repeats occurs without disruption of the SH3 domain, the latter should be accessible and functional. These results support a force-sensing and a plakin-domain-stabilizing role of the unique SH3 insertion, which puts plectin and the whole plakin family of proteins up front as a new class of potential mechanosensors [27]. As plectin is capable of multiple interactions with cytoskeleton constituents and can act as a sequestering and scaffolding platform for signaling molecules [5,6,29,30,31,32], it may play a central role in mechanosensing and launching of the related signaling cascades.

Another interesting structural feature of plectin is the relative high number of cysteines (17 in humans, 13 in rat) that are present in its *C*-terminal IF-interacting domain. On the basis of homology modeling and biochemical analyses of recombinant subdomains, a model was developed where disulfide bridges within the *C*-terminal globular domain were predicted to be partially responsible for the packing of its six submodules into the compact globe visualized by electron microscopy [33,34,35]. Hence, reversible disulfide bridge formation, including *S*-glutathionylation of cryptic cysteines, as reported, for example, for sarcomeric titin [36,37], could be another mechanism to modulate plectin’s elasticity.

Even the ~200 nm long α-helical rod domain of plectin may contribute to the elasticity of the molecule. IF networks and single IFs display elastic and strain-stiffening properties [20], with single IFs surviving more than threefold stretching without breakage [38,39]. Force–strain measurements of optically trapped vimentin filaments suggested that their resistance to breakage is attributable to the elastic stretching of α-helices followed by the uncoiling of α-helices into β-sheets, reflecting their ability to absorb energy; a strong stiffening observed at high strains has been proposed to result from increased pulling on the β-sheets [17,18]. As measured by cyclic filament stretching and relaxation, single filaments can dissipate more than 70% of the input energy [19]. It is possible that similar mechanisms apply to the α-helical rod domain of plectin, which, like IF proteins, dimerizes to form an α-helical coiled-coil and oligomerizes presumably via antiparallel lateral association of the dimers [14,40,41]. Force–strain measurements of single plectin molecules may clarify whether plectin as a filament interlinking and anchoring protein contributes to the viscoelasticity of IF networks in this way.

## 3. Plectin Mechanically Stabilizes IF Networks and Their Docking Platforms

In the absence of plectin, IF networks become fragile and less stress-resistant, as shown for cells expressing different types of IFs. Plectin-null keratin K5/K14-expressing dermal keratinocytes show a higher sensitivity to hypo-osmotic shock and okadaic acid (OA)-induced hyperphosphorylation, manifesting as faster network collapse [31,40]. Vimentin networks in plectin-null fibroblasts show a similar behavior when subjected to OA or heat-shock stress tests [42], as do K8/K18 networks in hepatocytes and cholangiocytes [43]. In accordance, IFs turned out to be less stable and consequently more soluble upon extraction from plectin-null cells [31,42]. Plectin deficiency leads also to increased susceptibility of IF networks toward oxidative stress-induced collapse, as demonstrated for vimentin IFs of renal endothelial cells upon *S*-nitrosylation [34]. The most obvious manifestation of IF network integrity to be dependent on plectin is the spontaneous collapse and aggregation of desmin networks, observed in myofibers from plectin knockout (KO) mice [13,44,45,46] and patients suffering from myofibrillar myopathies [16,47]. Intriguingly, the analyses of a series of distinct plectin KO mouse models in parallel enabled a spatial correlation of plectin isoform targeting sites with locally restricted IF disruption [13,44,46]. The mechanism of filament collapse remains obscure. Possible scenarios include unanchored filaments featuring less tension to undergo conformational changes that make them more accessible to proteases or modifying enzymes triggering filament disassembly, such as phosphorylation. In fact, an increased diameter of single vimentin filaments in plectin-null fibroblasts has been reported [42]. Furthermore, in the absence of IF crosslinking and docking, IF networks are likely to lose their compactness and become more vulnerable to uncontrolled local actomyosin-driven forces leading to their disorganization and collapse.

Devastating effects of plectin disruption on IF-docking site and local environment integrity have been described for several cases, the best studied of which are HDs of basal keratinocytes and the dystrophin glycoprotein complex (DGC) of myofibers. The challenge to HD integrity through dysfunctional plectin is reflected by the severe skin blistering phenotype of patients [16] and of knock-in and knockout mouse models [14], as well as by the disruption of HD precursor versions (type II HDs) forming ex vivo in diverse cell culture models [31,40,48,49,50].

In skeletal muscle myofibers, which enable monitoring of different isoforms and their docking platforms side by side in single polynucleated giant cells, the lack of plectin isoform 1f (P1f), the major linking element between the subsarcolemmal protein skeleton and the desmin IF network, leads to drastic structural alterations. These include partial separation of the contractile apparatus from the sarcolemma and massive dislocation of costameric protein complex constituents, such as β-dystroglycan (β-DG), dystrophin, neuronal nitric oxide synthase (NOS), syntrophin, and others [13]. In addition, it leads to disruption of NMJ integrity, including the loss of membrane infoldings and the dispersion AcChR clusters within the membrane [44]. In the cell interior, the specific disruption of Z-disc-associated P1d leads to dysfunction of a not yet characterized sarcomeric IF-docking platform. The existence of such a platform is implicit from studies showing similar sarcomeric phenotype manifestations of specifically isoform P1d-deficient and desmin-null mice, particularly disarrayed discs combined with misaligned myofibrils [13,51]. The disruptive effects of dysfunctional plectin isoforms on interior IF docking sites are also reflected by the dispersion of P1′s nuclear binding partner endophilin B2 over the entire nuclear surface in P1-deficient myofibers, contrasting its predominantly equatorial location in P1-expressing cells (see model shown in [46]). The docking station components of P1b, which is targeted to the mitochondrial membrane via its isoform-specific signal-anchor domain, still need to be characterized. Since P1b-mediated linkage of mitochondria to IFs has been shown to lead to mitochondrial fusion in myofibers and fibroblasts, with evidence for involvement of fusion-related protein mitofusin-2 [52,53], it would not be unexpected if a mitochondrial IF-binding platform contains components of the mitochondrial fusion/fission machinery or is linked to it. An IF docking platform of this kind could play an important role in mechanotransduction, especially as P1b-mediated IF–mitochondrion linkage is isoform-specific and affects mitochondrial respiratory functions [53].

In myelinating Schwann cells, β-DG, the core component of the DGC in Cajal bands, is crucial for the compartmentalization and stabilization of the myelin sheath. Similar to muscle, it was found that plectin (probably P1f) mediates the recruitment of vimentin IFs to the complex. Disruption of this association through Schwann cell-restricted plectin depletion in mice leads to the destabilization of the complex combined with increased myelin sheath deformations in peripheral nerves, as observed during aging of the animal [54].

Focal adhesions (FAs) located at the ends of contractile actomyosin stress fibers form tight connections between migrating cells and their underlying extracellular matrix. Monitoring the turnover of FAs in migrating single primary fibroblasts, a strikingly reduced lifetime of vimentin-associated (central) versus not IF-associated (peripheral) FAs was observed, indicating that plectin (P1f)-mediated recruitment of vimentin networks to FAs leads to their stabilization [55]. Similar observations were reported for migrating astrocytes [56].

## 4. Modulation and Compartmentalization of the Actomyosin Machinery through Plectin-Mediated IF Network Recruitment

### 4.1. Fibroblasts and Keratinocytes

By now, there is plenty of evidence that plectin mediates the interaction of IF networks with the actomyosin machinery. This idea has its roots in early immunofluorescence and interference microscopy studies that demonstrated co-distribution of plectin with adherens-type junctions in smooth muscle and focal contact adhesion plaques and actin stress fibers in fibroblast and glioma cells [57,58]. First evidence for a role of plectin in regulating actin filament dynamics was established in studies with primary fibroblasts and astroglial cells derived from plectin-deficient KO mice [59,60]. Not only was the actin–FA cytoskeleton in KO fibroblasts more extensively developed compared to plectin-expressing cells, but it also failed to show the characteristic short-term rearrangements in response to extracellular stimuli activating Rho/Rac/cdc42 signaling cascades. Alterations in actin-dependent processes, including motility, adherence, shear stress resistance, and morphology of cells, were the consequence [60]. Similarly, astroglial cells lacking plectin showed delayed morphological differentiation (stellation) upon cAMP stimulation, a process involving actin-based mechanisms mediated by the GTPase Rho. In this paper, it was also demonstrated that plectin’s ABD was functional and modulable by phosphatidylinositol bisphosphate (PIP_2_). Upregulation of FAs and stress fiber formation in response to plectin deficiency turned out to be a general phenomenon, applying also to basal dermal keratinocytes [31,50], endothelial cells [61], and epithelial monolayer cells. A significant increase in actin stress fibers could also be demonstrated by inducing the disassembly of IFs with either OA [31] or acrylamide [61]. Interestingly, in one of the first studies testing the functional diversity of plectin isoforms, a couple of isoforms showed conspicuous recruitment to stress fibers upon forced expression in cultured cells, either along the entire length of the fibers (P1a) or at their presumably most strained ends (P1f) [12].

A first, a more systematic study on plectin-mediated interactions between IF networks and the actomyosin cytoskeleton was carried out with primary and immortalized (keratin K5/K14-expressing) basal keratinocytes [31]. The results were consistent with a model where a compact, nucleus-encapsulating and HD (integrin β4)-anchored P1a/keratin cytoskeleton sequesters signaling molecules involved in stress response and migration, consequently having a suppressive impact on actomyosin-generated forces. Without P1a-mediated anchorage, actomyosin forces are set free, phenotypically manifesting as a significantly faster migration potential of plectin-null compared to WT keratinocytes. Here, the idea was born that plectin, via its HD-associated isoform [50], and in conjunction with keratin filaments, acted as a modulator of actomyosin forces driving migration [6,31]. This concept was confirmed by subsequent studies in which plectin’s role in keratinocyte migration and adhesion was assessed, either by addressing P1a directly [40,62] or plectin without isoform discrimination [49]. The distinction of isoforms is important in this context, because of evidence showing that the migratory behavior of keratinocytes can also be affected by P1c through a mechanism that appears to be different from that involving P1a [63]. The essential role of plectin as a mediator of IF network–actomyosin cytoskeleton interactions was confirmed in a recent study showing a mechanical coupling of HDs and FA complexes via plectin/IF networks in keratinocyte cell lines [49].

Important mechanistic insights into how plectin mediates the interplay between IF networks and the migration-driving actomyosin machinery were gained in studies on vimentin-expressing fibroblasts. Studying primary and immortalized plectin-null and wild-type cells, Burgstaller et al. [55] showed that mature FAs and their derivative fibronectin fibril-aligned fibrillar adhesions (FbAs) serve as docking sites for vimentin IFs in a plectin-, specifically P1f-dependent manner. Time-lapse video microscopy revealed that FA-associated P1f captures mobile vimentin filament precursors, which then serve as seeds for de novo IF network formation via end-to-end fusion with other mobile precursors. As filament precursors were also found to be associated with plectin isoforms other than P1f (e.g., P1c), the so-achieved elongation of filaments led to an IF network decorated with different plectin isoforms, increasing its crosslinking potential. Interestingly, vimentin filament precursor formation was found to be plectin-dependent, suggesting its physiological relevance [55,64]. P1f-mediated IF network formation at FbAs was shown to create a resilient cage-like core structure that encases and positions the nucleus while being stably connected to the exterior of the cell. The formation of this rather immobile IF network was suggested to act as a kind of physical constraint compartmentalizing the actomyosin machinery to the region of the advancing lamellipodium of migrating cells, thereby affecting cell shape and cell polarization. Schematic working models based on these studies were shown in [4,6,55].

A follow-up study with fibroblasts demonstrated that plectin, by integrating FAs into the IF network, modulates their function as integrin-based mechanosensing devices of cells. The authors proposed that the physical constraint provided by the FA and nuclear cage-anchored IF network leads to increased tension within the actomyosin system and modulation of G proteins and tension-sensor focal adhesion kinase (FAK) [42]. The hyperelasticity and rubber-like properties of the vimentin IF network [23] provide a fitting explanation for the mechanics of the postulated mechanical strain.

Specific targeting mechanisms of P1a and P1f to HDs and FAs, respectively, remain to be uncovered. The observation that truncated versions of P1a, lacking its *C*-terminal IF binding domain, are not recruited to integrin β4 [12,50], but only full-length versions, may be taken as an indication that the partnership of P1a with IFs is required for HD targeting; a similar suggestion was made on the basis of studies with keratin-depleted keratinocytes [65]. A recent study shed light on part of P1a’s head domain structure and provided insights into P1a-specific mechanisms regulating plectin-integrin β4 interaction in HDs via Ca^2+^-calmodulin (CaM) [66]. By resolving the crystal structure of the P1a-preceded ABD in complex with either the P1a-interacting domain of CaM or the plectin-interacting integrin β4 fibronectin domains, Song et al. [66] showed that one part of the intrinsically disordered isoform-specific head domain (amino-acid residues 23–37) of P1a is converted by CaM binding to an α-helix which relocates CaM to displace integrin β4 by steric repulsion. The results confirmed a molecular mechanism underlying Ca^2+^-CaM regulation of the P1a/integrin β4 and P1a/F-actin interactions via shunting the integrin β4 and F-actin from the complex with P1a. The 22 residue long isoform P1a-specific domain preceding the CaM binding site remains intrinsically disordered after CaM binding, making it a prime candidate for harboring a signal for targeting P1a either to integrin β4 domains other than the fibronectin domains or to a site near HDs, facilitating the local coupling of plectin to integrin β4 without the involvement of its isoform-specific domain; alternatively, it may harbor a signal affecting plectin’s *C*-terminal integrin β4-binding domain [67].

Considering P1f’s recruitment to FAs and FA-related structures [13,55], it would not come as a surprise if P1f was found to specifically interact with strained conformations of mechanosensitive proteins, such as talin, vinculin, tensin, α-catenin, and actinins, their associated proteins, or even components of the actomyosin machinery itself. Working models of this kind have been published [55], and some early observations would be consistent with this notion. For example, in their original electron microscopy study showing plectin sidearms extending from vimentin filaments in actin-depleted fibroblast cytoskeletons, Svitkina et al. [68] observed plectin-mediated interactions of IFs with actin stress fiber remnants that survived actin depletion by gelsolin and tentatively identified them as myosin II minifilaments. Moreover, when fluorescence-tagged versions of *N*-terminal fragments of P1f and P1a were ectopically expressed in cells, they showed contrasting association patterns with actin stress fibers. Whereas P1a fragments decorated stress fibers over their entire lengths, except for their FA-associated ends, P1f fragments (and full-length P1f) preferentially associated with vinculin-positive stress fibers endings. Furthermore, in cells overexpressing *N*-terminal P1f fragments, more actin seemed to be recruited to FACs as compared to untransfected cells or cells transfected with corresponding P1a fragments [12].

### 4.2. Endothelial Cells

That plectin-controlled IF cytoarchitecture and compartmentalization have a strong influence on the cellular actomyosin machinery was also demonstrated for vascular blood barrier-forming endothelial cells [61]. In this type of cell, plectin deficiency-inflicted perturbations of the vimentin cytoskeleton causes severe distortions of adherens junctions (AJs), as well as tight junctions, accompanied by the upregulation of radial actin stress fibers and loss of cortical AJ-associated actin structures. The reorganization of the actomyosin cytoskeleton led to a dramatic increase in internal traction forces, manifesting as higher contractility of adhering cells and a consequently decreased barrier function of cells. This study not only opened a new perspective on cytoskeleton-controlled vascular permeability, but also clearly demonstrated that plectin-organized IF scaffolds keep actomyosin contractility in check, thereby maintaining AJ homeostasis (see model shown by Osmanagic-Myers et al. [61]).

P1a is one of the major isoforms expressed in endothelial cells and, in phenotype rescue experiments, it showed a strong potential to restore AJ continuity in plectin-deficient cells [61]. Given the association of vimentin IFs with integrin α6β4 in endothelial cells [69] and recent progress in the identification of peripheral cortical IF structures in various cell systems [70], it is tempting to speculate that a cortical vimentin network tethered to integrin β4 by plectin (presumably P1a) plays a role in AJ homeostasis and mechanotransduction in this type of cell.

### 4.3. Neuromuscular Synapse

Plectin’s control over cortical actomyosin forces, executed through IF network recruitment to membrane-associated junctional complexes, is also reflected by the loss of NMJ integrity in plectin-deficient myofibers [44]. The formation and redistribution of acetylcholine receptor clusters forming the NMJ is known to be cortical actin-driven [71]. As assessed by live imaging, acetylcholine receptors (AChRs) in plectin-deficient myotubes were highly mobile and unable to coalesce into stable clusters, in contrast to their wild-type counterparts [44]. The disruption of the plectin/P1f-mediated linkages, that is, the uncoupling of AChRs and costameres from IFs, led not only to fragmentation and dispersion of AChRs within the sarcolemma, but also to the loss of the deep postsynaptic membrane infoldings that are characteristic of this type of synapse. In addition to deregulating the actomyosin machinery, the uncoupling of synaptic IFs impacts MT compartmentalization (see model shown by Mihailosvka et al. [44]).

It was shown that the head domain of isoform P1f in complex with the adjacent ABD specifically binds to the AChR scaffolding protein rapsyn, providing a mechanistic explanation for IF targeting to NMJs [44]. The data suggested that the exon 1f-encoded sequence, in contrast to other isoform-specific sequences, primes plectin’s ABD for binding to AChR cluster-associated rapsyn. The observation that agrin induced P1f–rapsyn binding speaks for the specificity of this interaction, as does the fact that ACF7/MACF, a cytolinker sharing high sequence homology of its *N*-terminal part with P1f, also shows binding to rapsyn [72]. Although the mechanisms of ABD-priming through plectin’s variable head domains remain obscure, by binding to other costameric proteins, such as fodrin and other DGC constituents, via domains outside of the targeting domain, P1f is likely to strengthen its IF recruitment potential, thereby consolidating the compactness and immobilization of the NMJ [44].

### 4.4. Mechanotransduction to the Nucleus

The question whether crosstalk between plectin–IF networks and the actomyosin machinery is contributing to mechanotransduction to the nucleus has been addressed in independent studies with keratin K5/K14-expressing basal keratinocytes [73] and with predominantly desmin-expressing myofibers [46], as representatives of cells from tissues that are most evidently affected in EBS-MD patients and corresponding mouse models. Studying the 3D nuclear morphology of keratinocytes cultured on micropatterned surfaces, Almeida et al. [73] found that the disruption of keratin network integrity through loss of plectin facilitated nuclear deformation in an actomyosin contractility-dependent manner. Due to the lack of evidence for a direct linkage of the keratin cytoskeleton with nesprin-3, a previously identified binding partner of plectin residing in the nuclear membrane of certain tissues [74], the authors suggested reduced filament crosslinking as a mechanistic explanation for reduced keratin filament density observed around the nucleus of plectin-deficient cells [73]. Investigating the plectin/keratin cytoskeleton response to alterations in matrix rigidity, plectin deficiency was found to promote mechanoresponsiveness and upregulation of lamin A/C, pointing toward an involvement of actin or MT responsive nesprin family members [75].

An alternative mechanism was revealed in studies on plectin’s role in nuclear mechanotransduction in myofibers, where isoform P1 was found to be preferentially associated with desmin filaments encapsulating myonuclei [13]. The phenotypic analysis of isoform P1-deficient mice showed that P1-mediated targeting of desmin IFs to myonuclei is essential for maintenance of the typically spheroidal architecture of myonuclei, as well as their proper positioning and movement along the myofiber [46]. Moreover, it was shown that P1 deficiency affects chromatin modifications and the expression of genes involved in signaling pathways mediating mechanotransduction, such as YAP, TAZ, STAT1, and STAT3 [46]. In light of recent studies revealing a protective function of IF networks shielding nuclei from actomyosin-driven mechanical deformations [76,77], the results obtained with myofibers suggest that plectin-mediated IF recruitment to the nuclear envelope is not only important for mechanotransduction, but also for preventing the actomyosin machinery from nuclear deformation.

Vanier et al. [78] showed that the isoform-specific head domain of P1 binds to nuclear envelope-associated endophilin B2, a protein of the Bin/Amphiphysin/Rvs (BAR) domain superfamily which is essential in controlling the shape and dynamics of intracellular membranes. P1 binds to endophilin B2 via a PETP motif within residues 150–153 in its isoform-specific head domain, thereby recruiting vimentin to the nuclear envelope of HeLa cells. The results suggested that the endophilin B2/P1 complex functions as a membrane-anchoring device organizing and stabilizing the perinuclear network of vimentin filaments [78]. The specific interaction of P1 with endophilin B2 was also established for myofibers [46].

Given the widespread expression of P1 in different tissues and types of cells [9,10,79], nuclear IF targeting through P1 can be expected to be a general mechanism applicable to cells expressing different types of IFs, including keratin networks. This does not exclude the possibility that other mechanisms, such as plectin-mediated or cytolinker-independent crosslinking of perinuclear IF networks [80], also play a role. In fact, the effectiveness of signaling through IF networks is likely to be based on a combination of a number of mechanisms, fine-tuned by the type of stress signals and composition of the mechanosensing machinery. In any case, the control of IF networks over the actomyosin filament system appears to depend heavily on plectin and its isoform-dependent location.

### 4.5. Invadopodia

There is evidence that the compartmentalization and control of the actomyosin machinery through plectin-mediated IF network recruitment plays an important role in cancer cell invasion and extravasation for metastasis. Plectin expression was found to be upregulated in many types of carcinoma cells and tumor tissues, leading to its use as prognostic biomarker and potential therapeutic target [15]. Tumor invasion, like basement membrane transmigration during embryonal development, relies on invadosomes, a collective term for invadopodia and podosomes. Sutoh Yoneyama et al. [81] showed that, in invasive bladder cancer cells, a prominent plectin–vimentin IF network provides invadosomes with a scaffold that facilitates cancer cell invasion and extravasation. As the knockdown of plectin in cancer cells severely impaired their potential to form invadopodia and reduced their capacities of extracellular matrix degradation, transendothelial migration, and metastasis, it was suggested that plectin-mediated crosslinking of vimentin scaffolds to actin stabilizes invadopodia and promotes their extension, which is critical for cancer cell invasion and extravasation for metastasis [81]. On the basis of this study and the previously demonstrated essential role of plectin/P1f-mediated IF network anchorage at FAs in 2D cell movement [55], a hypothetical model of invadopodium mechanics leading to protrusion into the 3D matrix was suggested [6]. According to this model, plectin-mediated anchorage of IFs at FA-like structures, which typically are clustered in a ring-shaped organization around the protrusions, leads to the formation of a dense, plug-like, locally immobilized structure at the dorsal side of the protrusion, which gains additional reinforcement by plectin crosslinks within the network. The IF network, stabilized and immobilized in this way, was suggested to provide the local physical constraint required for actomyosin-driven force generation toward the protruding front [6]. The elasticity and ability of IF networks to absorb and neutralize actomyosin-generated forces would explain the mechanics of this constraint [23]. Fitting this model would be the recently revealed modular actin nano-architecture enabling podosome protrusion and mechanosensing [82]. It was shown that the podosome protrusive core contains a central branched actin module encased by a linear, partially myosin II-crosslinked, contractile actin module, which consists of ventral filaments bound by vinculin and connected to the plasma membrane. On matrices enabling penetration, the vinculin-bound ventral actin filaments shorten, resulting in short-range connectivity and a focally protrusive state [83]. The two invadopodium protrusion models combined (Figure 1 in [6] and Figure 3f in [82], respectively) allow one to predict that the contractile ventral (and interpodosomal) actomyosin machineries lead to the recruitment of counteracting plectin-vimentin networks via mechanisms similar to those prevailing at single FAs and costameres.

Similar scenarios could be applicable to other types of highly migratory and invasive cancer cells. In fact, previous studies have shown that IFs play a role in stabilizing invadopodia [84], and McInroy and Määttä [85] described that plectin is targeted to podosome-like adhesions in SW480 colon carcinoma cells, regulating their invasiveness. Moreover, the expression of plectin, like that of vimentin, is upregulated in different types of carcinoma cells and tumor tissues, and curiously, plectin, has been localized on the extracellular surface of pancreatic ductal adenocarcinoma and other cancer cells [15]. In particular, it was shown that the plasma membrane-associated plectin isoforms P1a and P1f, together with integrin ß4, translocate and emanate from the cell surface via exosome trafficking, promoting pancreatic tumor growth and progression to an aggressive phenotype [86]. Since a symbiotic relation between invadopodia and exosome secretion resulting in increased invasion of cancer cells has been identified [87], plectin-mediated IF–actomyosin interplay could constitute an important regulatory element in tumor progression.

### 4.6. Mitosis

An interesting study bearing on plectin-mediated compartmentalization and control of actomyosin networks through vimentin filament networks was recently published by Serres et al. [88]. Addressing the question of how the cortex tension-regulating cortical actomyosin network is organized during mitosis, the authors identified vimentin and plectin as regulators of cortex architecture in the HeLa cell model. Using F-actin interactome mass spectrometry and super-resolution microscopy, it was found that, in mitotic cells, plectin recruits a network of thick vimentin cables to the cortex, which results in thinning of the cortical actin layer and contributes to increasing cortical tension in a plectin-dependent manner. When vimentin-deficient cells, confined by conditions mimicking the environment of a tissue were examined, they displayed increased occurrences of chromosome missegregation and multipolar spindles [88]. It will be of interest to assess whether mitotic mechanisms of this kind apply preferentially to rapidly growing transformed cell lines, such as HeLa cells, or to primary cells as well. Interestingly, aberrant mitotic spindle formation was also reported to occur spontaneously with high frequency in primary basal keratinocytes specifically lacking isoform P1c [63]. Whether P1c or other isoforms of plectin expressed in HeLa cells are responsible for compartmentalization and compacting of cortical actin during mitosis remains open; an involvement of other variants, including those shown to mediate IF recruitment to peripheral actomyosin machineries, such as P1f or P1a, would not be unexpected. In this context, it is noteworthy that fibroblasts, a cell type showing only partial disassembly of its perinuclear cage-like vimentin networks during mitosis, proceed faster through M-phase in the absence of plectin, with consequences for the size and IF content of daughter cells [64]. Hence, it is tempting to speculate that plectin controls peripheral and perinuclear IF networks during mitosis independently from each other and possibly via different isoforms and mechanisms.

## 5. The Microtubule Connection

The potential of plectin to interact with MTs was initially demonstrated using various experimental approaches, including co-assembly of plectin with MTs polymerized in vitro from cultured cells [89], solid-phase in vitro binding of purified (native) plectin to MT-associated proteins (MAPs) [90], visualization of plectin sidearms mediating IF–MT interaction in epithelial and fibroblast cells [68], colocalization of isoform P1c with MTs in keratinocytes [50], and MT targeting of P1c overexpressed in cell cultures [12].

The first more in-depth study on the impact that plectin and its associated IF networks have on MT functions was focused on basal keratinocytes, where P1c is expressed at the second-highest level after P1a, while it becomes dominant in suprabasal layer cells [50]. Investigating primary basal keratinocytes derived from isoform P1c-deficient (P1c^−/−^) mice in parallel with immortalized plectin-null (P0) cells of the same type, it was found that MTs in P1c^−/−^/P0 keratinocytes are more resistant toward nocodazole-induced disassembly and display increased acetylation [63]. In addition, live imaging of MTs in P1c^−/−^/P0 cells revealed altered MT dynamics, manifesting as the failure of MTs to undergo rapid disassembly (catastrophe) after reaching the cell margins, a trait that is typical for MTs in normal cells; instead, MTs in mutant cells continued to grow, undergoing bending at the periphery. On the basis of these observations, MTs in P1c^−/−^/P0 keratinocytes were suggested to be more resistant toward disassembly and, thus, more stable compared with their wild-type counterparts. The observation that phenotypes, such as decreased nocodazole sensitivity and increased acetylation of MTs, could be rescued by forced expression of the full-length, but not truncated version of P1c (without *C*-terminal IFBD) not only established direct causality between P1c loss and MT alterations, but also suggested that P1c was able to affect MTs only in partnership with IFs. Furthermore, MAP2 and tau protein expression in keratinocytes was confirmed by RT-PCR and immunostaining, and a considerable increase of MT-associated MAP2 was found by high-resolution microscopy in live P1c^−/−^ keratinocytes. On the basis of this and biochemical assays using recombinant proteins, a mechanistic working model for MT regulation through IF-bound P1c was presented [63]. According to this model, the binding of P1c to MTs antagonizes the MT-stabilizing and assembly-promoting function of MAPs through an inhibitory function exerted by plectin’s SH3 domain. On the cellular level, P1c deficiency led to changes in cell shape, increased velocity (but loss of directionality) of migration, smaller-sized FAs, higher glucose uptake, and mitotic spindle aberrations combined with reduced growth rates, strongly suggesting a control function of isoform P1c in keratin filament–MT crosstalk [63].

P1c-mediated crosstalk between IFs and MTs has also been shown to contribute to the regulation of MT-dependent functions in neurons of the central and peripheral nervous systems [91], where P1c expression is dominant over other isoforms [9,11]. The analysis of P1c-deficient (P1c^−/−^) primary dorsal root ganglion (DRG) and plectin-null (P0) hippocampal neurons, as well as P0 brain tissues, revealed that P1c deficiency phenotypically manifests with substantially elevated levels of MT-associated tau protein. Since several other genuine neural MAPs tested did not exhibit this phenotype, accumulation on axonal MTs appears to be specific for tau. The monitoring of MT growth in axons by video microscopy of EB3, an MT plus end tracking protein (+TIP), revealed a higher number of growing MT ends and a faster growth rate of MTs in P1c-deficient compared to WT neurons, indicating that P1c-mediated neurofilament–MT interactions affect the dynamic behavior of axonal MTs. As a result, a number of fundamental neurite functions were found to be compromised upon P1c deficiency, including neurite branching and translocation of vesicles and mitochondria.

In living cells, especially in neuronal axons, a proportion of the polymerized MT pool is stabilized, while the labile fraction of MT fibers displays rapid dynamics [92]. Qiang et al. [93] recently showed that MAP6, a genuine MT stabilizer, is enriched along the stable domains of MTs, while tau is associated with their more labile domains. Based on this and other evidence, tau was suggested not to act as a stabilizer of axonal MTs, as presumed in many previous studies, but rather as a provider of longer labile domains, thereby promoting MT assembly and concomitantly limiting the binding of stabilizers such as MAP6 [94]. Interestingly, P1c deficiency in axons phenotypically manifested very similar to MAP6 depletion, but conversely to tau depletion [91,93]. Thus, the P1c deficiency phenotype is consistent with the newly suggested role of tau as a labile MT domain provider.

**Figure 2:**
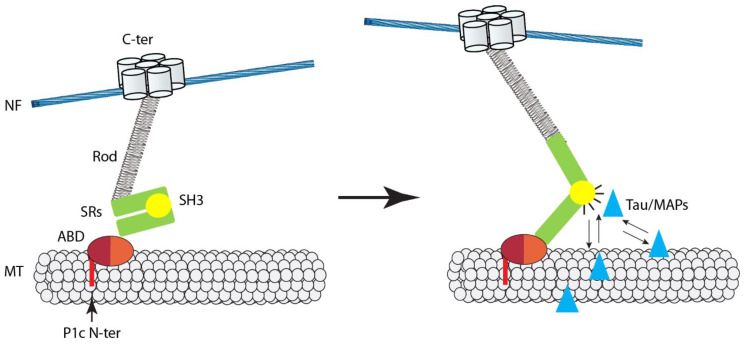
Hypothetical model depicting isoform P1c as a mechanosensitive regulator of MT dynamics in neurons. Binding of NF-associated P1c to MTs occurs via its isoform-specific *N*-terminal sequence (P1c *N*-ter) and the adjoining ABD; note: the ABD of the P1c variant expressed in neurons is of the 2α/3α type (see text). Left, without strain, the SH3 domain, embedded within the spectrin repeats (SRs) of the plakin domain, is autoinhibited (i.e., inaccessible to binding proteins) through its internal interaction with SR4. Right, on mechanical force (central large arrow), SRs partially unfold, exposing the SH3 domain on the exterior of the molecule and making it accessible to interacting proteins, such as tau and other MT regulators. Such interactions may lead to alterations in MT dynamics in response to strain. (For details see text).

According to the working model shown in Figure 2, P1c associates with axonal MTs via its isoform-specific tubulin-binding domain and participates in the regulation of MAP–MT interactions by binding to MAPs via its plakin domain-embedded SH3 domain. Theoretically, this could have positive or negative effects on MT stability/dynamics, depending on the type of MAP that becomes subject of P1c regulation. In the case of axons, the depletion of P1c led to an accumulation specifically of tau on MTs, which, consistent with its newly suggested role, promotes MT dynamics, manifesting as increased MT growth and assembly rate. In P1c-deficient keratinocytes, the observed increase in MT stability was accompanied by increased MAP2 decoration of MTs in cells [63], while, in P1c^−/−^ neurons, no increase in MT-bound MAP2 was found [91]. This would be consistent with the notion that, dependent on IF type and cellular settings, P1c-mediated IF interactions differentially affect MT network properties. Further studies are required to understand the mechanics of these interactions in more detail. In particular, it will be of interest to investigate whether the putative force-sensing ability of plectin’s SH3 domain, which is likely to regulate its accessibility for binding partners such as MAPs and tau, plays a role in axonal mechanotransduction.

Alterations in MT dynamics elicited by plectin deficiency have also been reported for myofibers, where desmin constitutes the major IF system. In a study addressing physiological consequences of plectin accumulation observed at the sarcolemma of muscular dystrophy patients and mdx mice [95], the phenotyping of different plectin KO mouse lines in parallel revealed that, in subsarcolemmal regions of plectin-null myofibers, MT networks were more prominent, appearing more robust, compared to their wild-type counterparts. The MT networks were also more resistant toward nocodazole-triggered disassembly, indicating higher stability. Interestingly, similar to keratinocytes and neurons, the levels of MT-bound MAPs were found to be increased, as demonstrated for the 120 kDa (high-molecular-weight) variant of tau, one of the main MAPs expressed in muscle [96], as well as MAP4. This also supports the notion that plectin, by bridging MT and IF networks, participates in regulating MT–MAP association with consequences for cellular functions involving MT dynamics. Indeed, it was shown that plectin (P1f) levels affect the translocation of glucose transporter GLUT4, an MT-dependent process affecting glucose uptake [95]. Moreover, the disruption of plectin-mediated IF–NMJ linkages in myofibers, leading to a dramatic restructuring of the postsynaptic endplate and its microenvironment, entails a massive invasion of MTs into the area [44].

Regarding targeting mechanisms, previous studies have indicated that P1c targets MT polymers via direct binding to tubulin and independently of MAPs. Binding requires the P1c-specific head domain joined to the ABD. When a series of recombinant *N*-terminal fragments of plectin variants was subjected to co-sedimentation assays with MTs assembled from purified (MAP-free) tubulin, a P1c variant with an ABD version containing additional short sequences of five and 12 amino-acid residues (encoded by alternatively spliced exons 2α and 3α [9]) showed optimal MT binding [91]. Intriguingly, this P1c variant is exclusively expressed in nerve tissues [9]. However, binding of P1c to MTs is not restricted to this variant, as *N*-terminal fragments of P1c without these insertions also showed binding, albeit at reduced levels. Indeed, P1c associates with MTs in keratinocytes [63], a cell type that expresses exclusively P1c transcripts without 2α/3α-specific sequences. Thus, the combination of exon 1c with exons 2α/3α in P1c is not a strict requirement, but appears to present an ideal alliance in forming an optimal MT binding interface, a molecular feature that could be of particular importance for the fine-tuning of plectin-mediated IF–MT crosstalk in neurons. The structural analysis of tubulin–P1c complexes will probably be required to solve the mechanism underlying how P1c’s intrinsically unstructured head domain primes the adjoining ABD for tubulin binding and what role the 2α/3α exon splicing plays in this process.

## 6. Plectin and IF Networks as Mutually Dependent Partners

The most convincing evidence for the hypothesis that plectin and IFs act in partnership, with different, but apparently equally important functions, probably comes from the direct comparison of plectin and IF protein knockout phenotypes that mimic or are very similar to each other. However, the significance of such comparisons depends on how many IF network types, which set of plectin isoforms are expressed in the study object, and how efficiently they have been knocked out.

Vimentin-null fibroblasts, similar to plectin-null fibroblasts, exhibit enlarged FAs, prominent actin stress fibers, compromised motility, and delayed wound healing in mice, consistent with the partnership hypothesis [5,42,60,97,98]. Furthermore, studies in which experiments were performed with plectin-null and vimentin-null fibroblasts in parallel showed that uncoupling of the vimentin network from FAs, either by knockout of the network altogether or by disruption of its plectin-mediated anchorage, attenuates the mechanosensing ability of the cells in the same way, including FAK and RhoA modulation [42]. Impaired migration and increased FA and stress fiber formation are also characteristic for isoform P1-deficient fibroblasts [79].

Similar phenotypes were observed in studies where keratinocytes fully depleted of keratins were directly compared with plectin-null keratinocytes. The major phenotypes shared between these two types of mutant cells were enhanced migration and adhesion, as well as destabilization and enhanced endocytosis of their HD integrin α6β4-based docking sites [99]. Both types of cells exhibited also a similar phenotype regarding stiffness-dependent lamin A/C expression [75].

A mutually dependent partnership relation of plectin and IF network functions is particularly evident in the case of muscle fibers. The analysis of muscle tissues and single (teased) myofibers from a series of plectin knockout mouse lines, including totally depleted, specific isoform-depleted, and double KO lines, in parallel with the corresponding specimens from desmin KO mice, revealed largely similar phenotypes on all levels [13]. Examples comprise the misalignment and disorientation of myofibrils and Z-discs [13] (compare to [51]), aggregation, shape changes, location, and respiratory activity of mitochondria [13,53], the shape, spatial arrangement, and mobility of myonuclei [46], and the stabilization and rapid degradation of AChR clusters in myotubes [44]. These and other phenotypic similarities showed that desmin deficiency phenotypically closely resembles plectin deficiency, consistent with the notion that the loss of IFs and the disruption of their plectin-mediated interactions affect cell integrity in similar ways. The observation that some phenotypes, such as endplate deformation, manifest with lesser severity in desmin-deficient mouse muscle compared to muscle fibers depleted of all plectin isoforms is explainable by compensatory or supplementary effects of keratins K8/K19 or other types of IFs expressed in myofibers.

The conclusion that the loss of IFs and the disruption of their plectin-mediated interactions affect cell integrity in similar ways was also reached by de Pascalis et al. [56] in a study on the role of IFs in the collective migration of astrocytes. They showed that the depletion of IFs in astrocytes, by combined knockdown of vimentin, glial fibrillary acidic protein (GFAP), and nestin, decreases migration speed, persistence, and directionality, accompanied by a drastic change in actin organization. Like in fibroblasts [42,55], traction forces in migrating astrocytes were found to be exerted through FAs. The migratory phenotypes of IF-depleted astrocytes were mimicked by reducing plectin expression in primary astrocytes by siRNA, supporting the hypothesis that IFs regulate cell migration and actomyosin-generated traction forces through plectin-mediated interactions [56].

Studying a lung cancer cell line that shows upregulation of integrin α6/β4, but does not form HDs, Colburn and Jones [100] reported that plectin-mediated recruitment of vimentin filaments to integrin clusters leads to the accumulation of active Rac1 at the site, with effects on cell motility. Interestingly, the knockdown of either vimentin or plectin led to failed Rac1 clustering at α6β4 integrin complexes, again indicating that the docking of IFs via plectin is essential for their functionality, in this case, probably to act as a signaling hub regulating cell motility behavior.

A close resemblance of phenotypes, manifesting as reduced motor nerve conduction velocity and shift to axons with smaller calibers, was also observed for P1c-deficient and neurofilament heavy chain-null mice [11,101].

## 7. Conclusions, Working Model, and Perspectives

The central role of plectin as a mediator of IF functions has become increasingly clear in recent years. Mainly on the basis of phenotypic analyses of a series of tissue-restricted and isoform-specific knockout mouse lines and their cell derivatives, a diverse spectrum of functions attributed to IFs was shown to depend on the symbiotic interplay between IFs and plectin. Its universal and strong IF-binding ability and its isoform-dictated differential targeting and interaction capabilities enable plectin to physically connect IFs with diverse internal and peripheral membrane-associated cytoskeletal junctions. In concert with IF–IF interlinking through plectin molecules, the isoform-mediated anchorage of IFs at multiple sites leads to the formation of robust filamentous networks integrating the nucleus, mitochondria, plasma membrane surface receptors, and other junctional complexes. A network of this sort is ideally suited to act as a medium for mechanotransduction. Furthermore, the recently established ability of IFs to form viscoelastic networks that absorb energy, thereby counteracting actomyosin forces and MT penetration at junctional sites of the cytoskeleton [23], provides a mechanistic explanation for the modulation and physical constraint of the actomyosin machinery through IF networks, as observed in many cell systems.

**Figure 3:**
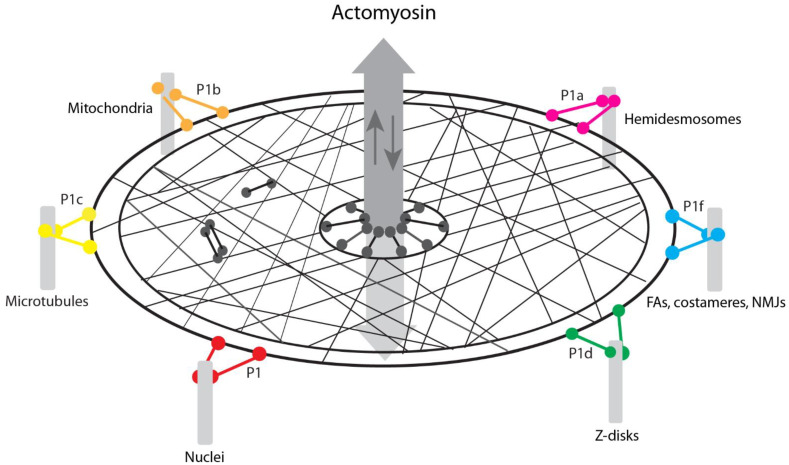
Model depicting plectin as a key element in the modulation of actomyosin forces through IF networks. The IF network is depicted as a safety net held up and spanned between posts via plectin sidearms. The posts represent the various organelles and cytoskeletal junctional sites that, in cells, are linked to IFs via different (color-coded) plectin isoforms. Note: desmosomes and other intercellular junctions, which most likely also are linked to IF networks via plectin, are not shown, as the type of isoform involved has yet to be identified. The bidirectional arrow in the center of the net stands for the actomyosin machinery which is physically linked to the IF network via plectin sidearms that presumably can be formed by any type of isoform as long as it contains a functional ABD (black dumbbells); plectin-mediated IF networking (exemplified by a dimeric and a tetrameric molecule) is also probably isoform type-independent as well. For hypothetical IF networking modes of dimeric and tetrameric plectin molecules, see [6]. Note: the depicted plectin-mediated linkages combined with the viscoelastic properties of the IF network (safety net) provide a mechanistic explanation how IFs modulate and counteract actomyosin-generated forces.

According to the working model depicted in Figure 3, one may look at the IF network as a safety net held up and spanned across posts via plectin molecules. In this illustration, the posts stand for the various organelles and junctional sites of the cytoskeleton that are linked to the network via different isoforms of plectin. Both the IF network and the plectin linkers are meant to have viscoelastic properties. Plectin-mediated IF networking and interactions with the actomyosin machinery may not necessarily require specific plectin isoforms, although some isoforms may be better suited for these functions than others. The local concentration of IFs in certain areas of the cytoplasm in response to actomyosin-generated forces and the ensuing buildup of physical constraints lead to modulation of the actomyosin machinery and its compartmentalization to regions where IF networks are less dense or unstrained due to not being anchored, such as at the leading edge of migrating cells [55]. The model predicts the distortion and collapse of IFs in local areas upon disruption of specific isoforms, as one observes in isoform knockout cells and tissues. Moreover, the model provides a testable mechanism for how MTs become integrated into the mechanosensing machinery of actin fibers and IFs, via plectin-mediated MAP regulation (see also Figure 2). As the functions and, in particular, any specific interaction partners of some of the plectin isoforms have not been studied and identified, it can be assumed that the model as presented in Figure 3 will be further complemented and extended in the near future. This applies especially to the integration of intercellular junctions, such as desmosomes and adherens junctions, into the model.

The validation of this concept will be a challenging task for future research, probably requiring more than one experimental approach. For example, the targeting mechanism and specific interaction potentials of some of the plectin isoforms are only partially understood. Targeting signals or site-specific interaction domains residing in isoform-specific head domains have been described for a number of isoforms, but only in the case of P1b was the head domain shown to suffice for site-specific targeting. In most other cases, the results reported point toward an involvement and combined action of the specific head domains with the adjacent ABD in determining targeting preferences. However, the mechanisms how different first exon-encoded protein domains “prime” the adjacent ABD or other parts of the molecule remain unexplained. Unraveling the targeting mechanism of isoform P1d will be of particular interest, as it accounts for roughly 50% of total plectin of myofibers, is exclusively associated with Z-discs, and is endowed with a head domain comprising just five amino-acid residues. To understand the isoform-dependent plasticity of plectin’s ABD and the conformational changes fostering binding to different partners, atomic-level structural analyses of plectin’s diverse *N* termini could provide useful information. Unfortunately, this approach is hampered by some of the isoform-specific domains having an intrinsically disordered structure [66].

Other open questions of interest are related to how the expression of isoforms is regulated on the transcript level. For instance, do individual isoforms or certain sets of isoforms have individual promoters, and is the expression of certain isoforms/sets of isoforms coupled to that of IF protein types, e.g., P1a and K5/K14, P1f/P1 and vimentin, or desmin and P1d? How are such patterns changed during development and differentiation of cells, or in epidermal–mesenchymal transition (EMT) of cancer cells? Interestingly, in this context, there is evidence for epigenetic regulation of plectin, as a risk locus for osteoarthritis has recently been identified in noncoding exons of the plectin gene [102,103]. Additional big challenges lying ahead are the unraveling of the molecular mechanisms behind plectin’s involvement in cancer progression and the exploration of plectin’s role in brain and peripheral nerve cell function (see related article in this Special Issue series). New insights along these lines should be helpful for a better understanding of the growing number of pathological conditions caused by dysfunctions of plectin and other cytolinker proteins, as well as IFs in general.

## Data Availability

This review does not present new experimental data. For data availability, please refer to the respective original papers.
